# Crystal structure of a Ca^2+^-dependent regulator of flagellar motility reveals the open-closed structural transition

**DOI:** 10.1038/s41598-018-19898-7

**Published:** 2018-01-31

**Authors:** Tomoki Shojima, Feng Hou, Yusuke Takahashi, Yoshitaka Matsumura, Masahiko Okai, Akira Nakamura, Katsutoshi Mizuno, Kazuo Inaba, Masaki Kojima, Takuya Miyakawa, Masaru Tanokura

**Affiliations:** 1 Department of Applied Biological Chemistry, Graduate School of Agricultural and Life Sciences, The University of Tokyo, 1-1-1 Yayoi, Bunkyo-ku, Tokyo 113-8657 Japan; 2 Laboratory of Bioinformatics, School of Life Sciences, Tokyo University of Pharmacy and Life Science, Hachioji, Japan; 3 Shimoda Marine Research Center, University of Tsukuba, Shizuoka, 415-0025 Japan

## Abstract

Sperm chemotaxis toward a chemoattractant is very important for the success of fertilization. Calaxin, a member of the neuronal calcium sensor protein family, directly acts on outer-arm dynein and regulates specific flagellar movement during sperm chemotaxis of ascidian, *Ciona intestinalis*. Here, we present the crystal structures of calaxin both in the open and closed states upon Ca^2+^ and Mg^2+^ binding. The crystal structures revealed that three of the four EF-hands of a calaxin molecule bound Ca^2+^ ions and that EF2 and EF3 played a critical role in the conformational transition between the open and closed states. The rotation of α7 and α8 helices induces a significant conformational change of a part of the α10 helix into the loop. The structural differences between the Ca^2+^- and Mg^2+^-bound forms indicates that EF3 in the closed state has a lower affinity for Mg^2+^, suggesting that calaxin tends to adopt the open state in Mg^2+^-bound form. SAXS data supports that Ca^2+^-binding causes the structural transition toward the closed state. The changes in the structural transition of the C-terminal domain may be required to bind outer-arm dynein. These results provide a novel mechanism for recognizing a target protein using a calcium sensor protein.

## Introduction

In many species, the female gamete or its associated structures release chemoattractants to attract spermatozoa. The chemoattractant gradient provides cues that guide sperm chemotaxis toward the egg^1^. In the sea urchin sperm, after binding of the chemoattractant peptide to its receptor, rapid synthesis of cGMP is induced to activate the K^+^-selective cyclic nucleotide-gate (KCNG) channel, leading to membrane potential (*E*_m_) hyperpolarization. The *E*_m_ change facilitates the Ca^2+^ extrusion activity of K^+^-dependent Na^+^/Ca^2+^ exchangers (NCKX) and results in an influx of Ca^2+^ into the cell^2,3^. This transient change in the intracellular Ca^2+^ concentration ([Ca^2+^]_i_) is necessary for the directional changes of the sperm flagellum^4–7^. Spermatozoa from marine invertebrates have been especially used to study the mechanism to control flagellar motility during sperm chemotaxis^8–11^. The spermatozoa of the ascidian *Ciona intestinalis* clearly exhibit chemotaxis toward the egg and a transient [Ca^2+^]_i_ increase in the flagellum accompanying a change of the swimming direction along a chemoattractant gradient field^12–14^. The sperm keeps straight swimming at lower [Ca^2+^]_i_, and higher [Ca^2+^]_i_ induces asymmetric flagellar bending for the turning motion^15,16^.

Ca^2+^ ions activate axonemal dyneins through the phosphorylation of the Txtcx-2-related light chain (LC) of outer-arm dynein and dephosphorylation of an intermediate chain (IC) of inner-arm dynein to induce a change in the flagellar beat, allowing the swimming of sperm along a gradient of the chemoattractant^17–20^. These responses are mediated by a sharp increase in [Ca^2+^]_i_ from 10^−6^ M to 10^−4^ M^21^. Studies on dynein-driven microtubule sliding in isolated axonemes have indicated that the calcium signal may be mediated by calmodulin and a calmodulin-dependent kinase^22^. The dynein light chain from the outer arm of *Chlamydomonas* flagella was also shown to contain a Ca^2+^-binding regulatory protein, which is directly associated with the γ dynein heavy chain^23^. However, the mechanism underlying the Ca^2+^-dependent regulation of dyneins remains obscure.

Recently, an axonemal Ca^2+^-binding protein from ascidian *C*. *intestinalis*, named calaxin, was demonstrated to bind to a heavy chain (HC) of outer-arm dynein and tubulin in a Ca^2+^-dependent manner and to be essential for the propagation of Ca^2+^-induced asymmetric flagellar bending^24,25^. Based on the amino acid sequence, calaxin belongs to the neuronal calcium sensor (NCS) protein family. Members of this protein family include recoverin and frequenin, which undergo conformational changes upon Ca^2+^ binding^26–29^, providing the surfaces to interact with their target proteins. To elucidate the relationship between the Ca^2+^-dependent regulatory mechanism and structural features of calaxin, we herein solved crystal structures of calaxin in the Ca^2+^-bound and Mg^2+^-bound forms. The crystal structure of calaxin shows an open-closed structural transition in both forms, and small-angle X-ray scattering data provides the evidence of structural transition in solution.

## Results

### Overall structure of calaxin in the Ca^2+^-bound form

**Figure 1:**
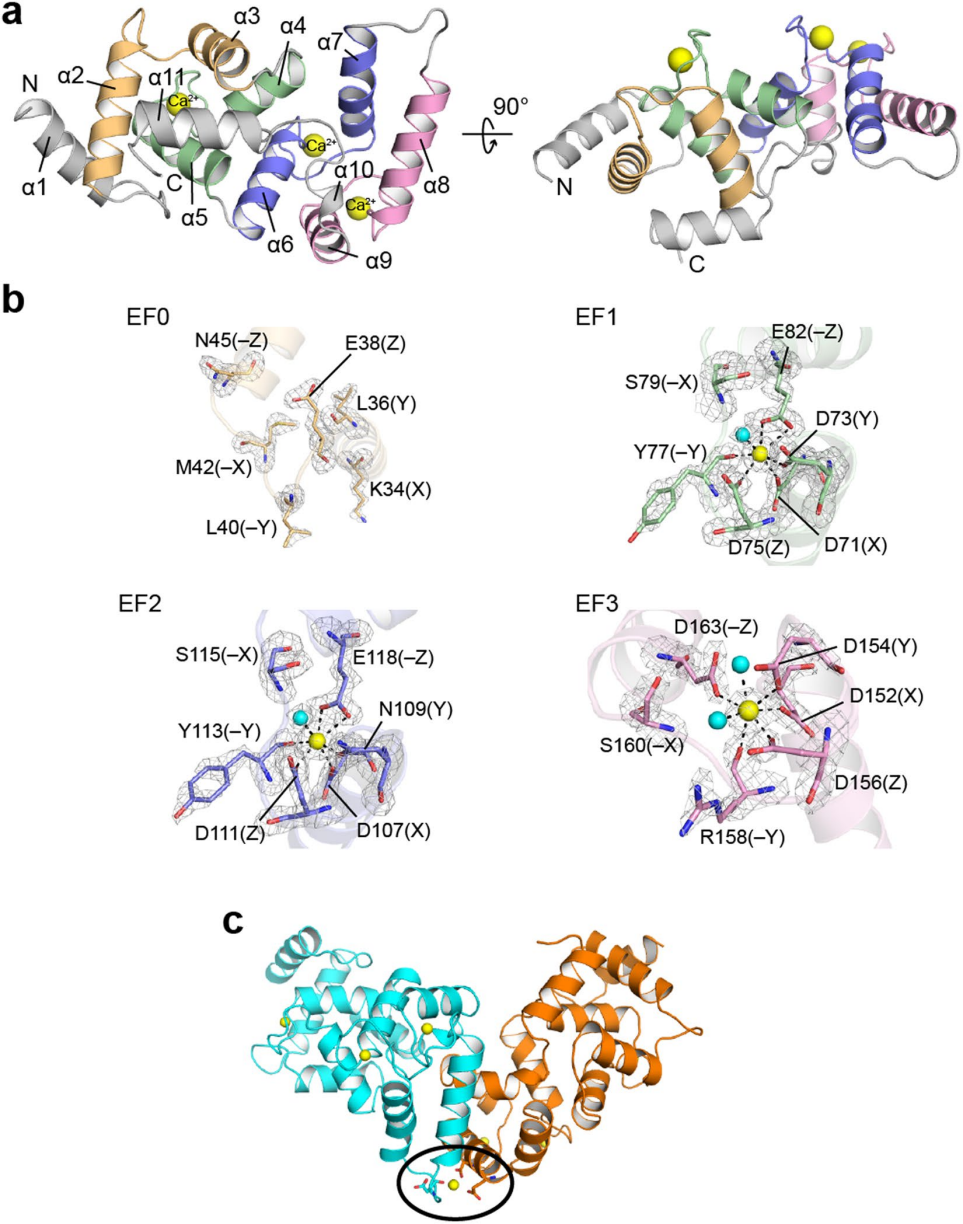
Structure of the Ca^2+^-bound form of calaxin. (**a**) Ribbon diagrams of the overall structure of calaxin in the Ca^2+^-bound open state. EF0, EF1, EF2 and EF3 are shown in light orange, pale green, slate and pink, respectively. Ca^2+^ ions are indicated by a yellow sphere model. (**b**) Close-up view of individual EF-hands binding a Ca^2+^ ion. Ca^2+^-binding residues are shown as a stick model. Ca^2+^ ions and water molecules are indicated by yellow and cyan spheres, respectively. The coordination bonds with Ca^2+^ ions are highlighted with dashed lines. The *F*_o_ − *F*_c_ omit maps of EF1, EF2 and EF3 are shown as gray meshes contoured at 3σ. (**c**) Calaxin dimer in the open state (cyan) and the closed state (orange) in an asymmetric unit. Ca^2+^ ions are represented as yellow spheres. Side chains coordinating Ca^2+^ between dimers are represented as sticks (surrounded by a black circle).

The crystal structure revealed that calaxin was composed of 11 helices (Fig. 1a and Supplementary Table 1) and contained four EF-hand motifs (Fig. 1b). Similarly to other NCS-family proteins, calaxin can be divided into two domains: the N-terminal domain and the C-terminal domain. In the N-terminal domain, EF0 (residues 23–55) interacted with EF1 (residues 60–92), and EF2 (residues 95–129) and EF3 (residues 137–170) formed the C-terminal domain (α6 through α10 helices) by interacting with each other. Both domains adopted their respective hydrophobic pockets, and the extended α11 helix was accommodated in the hydrophobic pocket of the N-terminal domain.

The classical EF-hand is characterized by a sequence of 12 residues involved in Ca^2+^ binding. In calaxin, EF1 and EF2 were composed of the conserved residues for Ca^2+^ binding that are generally observed as D, D/N, D/N and E at the positions X, Y, Z and −Z, respectively (Fig. 1b)^30^. These residues formed five coordinate bonds with Ca^2+^, including bidentate coordination by Glu. The position −Z of EF3 was substituted by Asp, which coordinates Ca^2+^ ion in a monodentate manner (Fig. 1b). In this type of EF-hand, the shorter side chain of Asp at the position −Z changes the angle of the canonical loop residue, resulting in a smaller and more compact Ca^2+^ binding motif^31^. By contrast, no characteristic residues are observed in EF0 (Supplementary Fig. 1), resulting in an impaired capacity to bind Ca^2+^ as shown in the crystal structure of calaxin (Fig. 1a and b). Among three Ca^2+^-bound EF hands, only EF2 has a Gly112 at the position between Asp111 (Z) and Tyr113 (−Y). The Gly residue of this position is known to be compatible to the bend of the loop region after position Z^32^, indicating that EF2 might allow its conformational changes upon Ca^2+^ binding (Fig. 1b and Supplementary Fig. 1). Moreover, we found that another Ca^2+^ ion is bonded between two calaxin molecules in an asymmetric unit (Fig. 1c). This Ca^2+^ coordination may contribute to stabilize the crystal packing.

### Conformational differences between two calaxin molecules in an asymmetric unit

**Figure 2:**
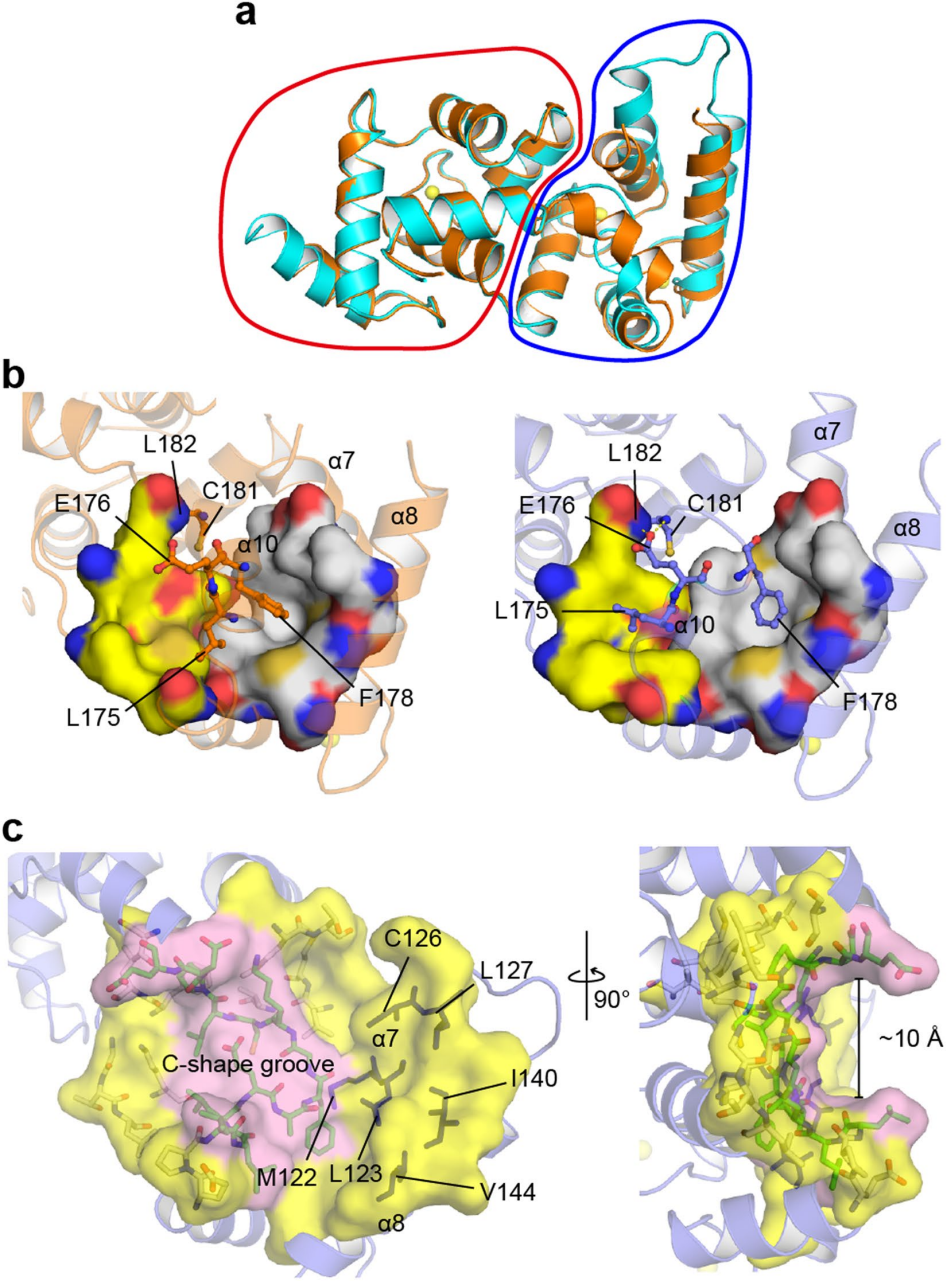
Structural basis for the open-closed transition of calaxin. (**a**) Structural comparison between the open state (cyan) and closed state (orange) in the Ca^2+^-bound form. The N-terminal domains and C-terminal domains are circled by a red line and a blue line, respectively. (**b**) Structures of Ca^2+^-bound calaxin in the closed (left) and open (right) states. Stick models indicate the residues critical for the twist of EF-hand motifs in the process of the open-closed transition. Hydrophobic holes for F178 and L175 binding are indicated by white and yellow surfaces, respectively. (**c**) Exposed surface of calaxin in the open state. The C-shaped groove (pink surface) is highlighted in pink, and residues forming the groove are colored green. Residues on the exposed surface and twisted helices are shown by magenta sticks and slate ribbon, respectively.

We found that the crystal structure adopts the different conformations in the C-terminal domains between two calaxin molecules in an asymmetric unit: One molecule is in the open state and the other is in the closed state (Fig. 2a). Compared with the open state, the closed state exhibited the inward movement of the α7 and α8 helices and reduced the exposure of the hydrophobic surface formed by the α7, α8 and α10 helices (Met122, Leu123, Cys126, Leu127, Ile140 and Val144) (Fig. 2b and c). Unlike the C-terminal domain, the N-terminal domain showed little structural difference between the open and closed states (Fig. 2a). Another conformational change was observed at the α10 helix. The residues (from Phe172 to Cys181), which formed a 3-turn helix in the closed state, relaxed the helical structure, resulting in a 1-turn helix and a longer loop between the α10 and α11 helices in the open state (Fig. 2b). The side chain of Phe178, which is located in the α10 helix, was accommodated in the hydrophobic surface formed by the α7, α8 and α10 helices in both the closed and open states. Phe178 was dragged by the movement of the α7 and α8 helices, which relaxed a part of the α10 helix in the conformational transition to the open state and induced a C-shaped groove with a width of ~10 Å (Fig. 2c). Furthermore, Glu176 also formed hydrogen bonds with the main-chain imino groups of Cys181 and Leu182 in the open state, resulting in the stabilization of the loop between the α10 and α11 helices in the open state. Two molecules in the open and closed states contacted each other using the α7 and α8 helices in the crystals (Supplementary Fig. 2). This contact may be due to crystal packing, resulting in the appearance of the open and closed states in an asymmetric unit. The simultaneous existence of both states in the Ca^2+^-bound form as crystal structures indicates that the driving force of Ca^2+^ binding to calaxin may be insufficient to move the α7 and α8 helices and induce the conformational change of the α10 helix.

### EF2 and EF3 play pivotal roles in the conformational change between the open and closed states

To characterize the conformational differences in the EF-hands, we measured the interhelical angles between the E-helix and F-helix in each EF-hand (Table 1). EF0 and EF1, which reside in the N-terminal domain, showed slight changes in the interhelical angle between the open and closed states. However, EF2 and EF3 induced a significant change in the interhelical angle in the transition from the closed state to open state, corresponding to the large conformational change in the C-terminal domain (Fig. 2a). This implies that EF2 and EF3 contribute to produce the driving force to convert between the open and closed states.

**Table 1:** Interhelical angle between the E-helix and F-helix in each EF-hand of Ca^2+^-bound calaxin.

EF-hand	open state (°)	closed state (°)	open state − closed state (°)
**EF0**	63.38	67.01	−3.63
**EF1**	58.01	61.82	−3.81
**EF2**	91.41	72.48	18.93
**EF3**	94.26	80.31	13.95

**Figure 3:**
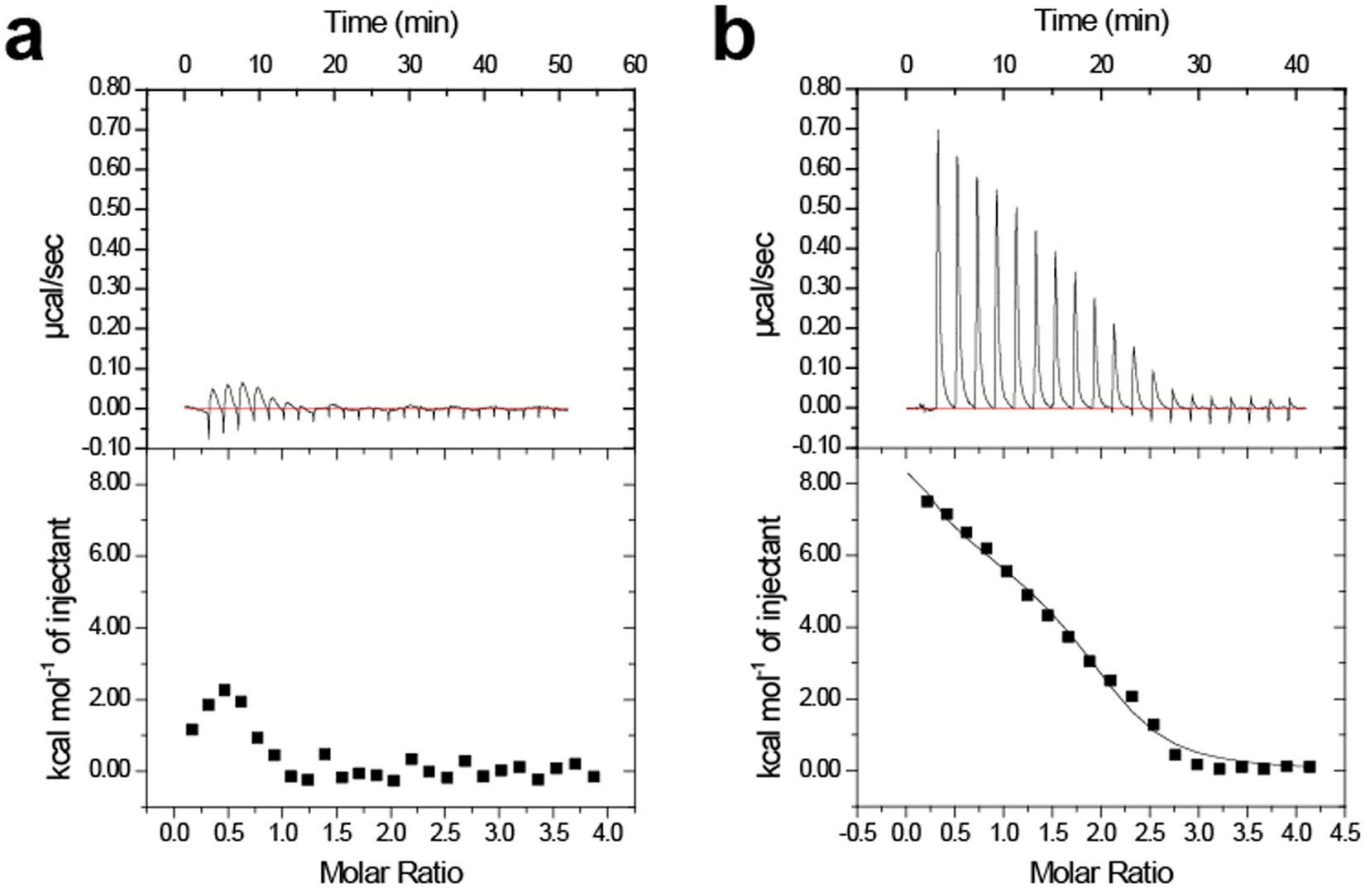
Isothermal titration calorimetry of Ca^2+^ binding to the EF2-defective E118A mutant (**a**) and EF3-defective D163A mutant (**b**).

To validate the importance of EF2 and EF3, we performed isothermal titration calorimetry experiments using two mutants, E118A and D163A (in the −Z position of EF2 and EF3, respectively) (Fig. 3). Some studies have reported that EF-hand proteins show large enthalpy changes by Ca^2+^ titration^33,34^. Regarding calaxin, it has previously been reported that Ca^2+^ binding to WT at 4 °C exhibits an endothermic enthalpy change^25^. E118A and D163A are predicted to be disabled for Ca^2+^ binding to each EF-hand. Ca^2+^ titrations to D163A represent endothermic binding similar to that of WT. However, Ca^2+^ binding to E118A showed a remarkably lower endothermic heat, indicating that the loss-of-function mutation in EF2 extinguishes the ability of the other EF-hand to bind Ca^2+^. The circular dichroism spectroscopy showed that both E118A and D163A mutants retained their secondary structures despite respective mutation (Supplementary Fig. 3). The results indicate that E118A mutation affects the sensitivity of calaxin to Ca^2+^ ions without the disruption of the native conformation.

### The α11 helix contributes to the thermostability of calaxin in the Ca^2+^-bound form by hydrophobic interaction

**Figure 4:**
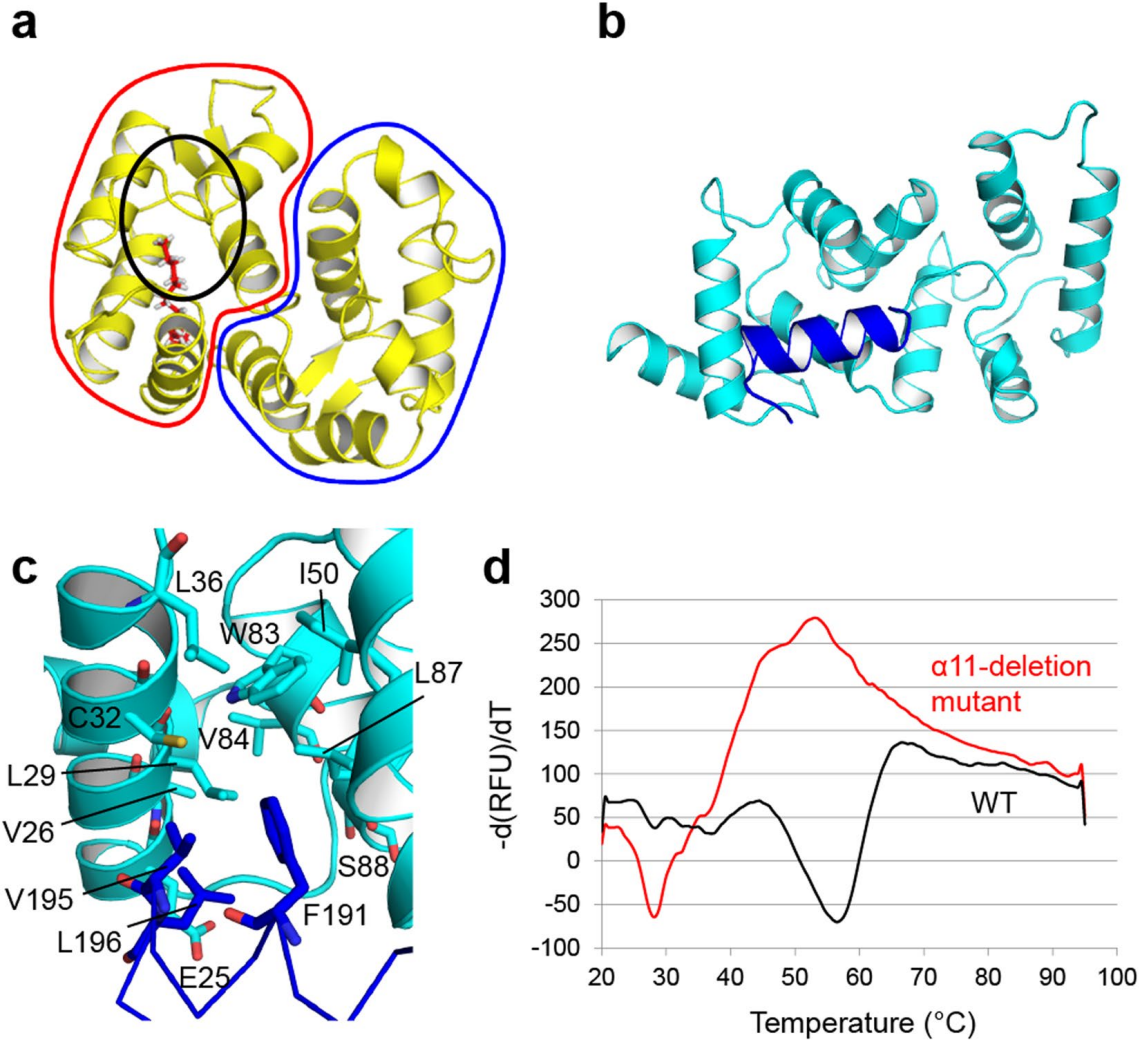
Contribution of the α11 helix to the thermostability of calaxin. (**a**) Solution structure of recoverin in the Ca^2+^-free state (PDB ID: 1IKU). The N-terminal domain and C-terminal domain are surrounded by a red line and a blue line, respectively. The N-terminal myristoyl group is depicted in red sticks. The black circle shows the hydrophobic pocket interacting with the myristoyl group. (**b**) Crystal structure of Ca^2+^-bound calaxin in the open state. The α11 helix is colored in blue. (**c**) Hydrophobic interaction between the C-terminal α11 helix and N-terminal domain. The α11 helix is depicted in blue lines, and its hydrophobic residues (F191, V195 and L196) are shown as blue sticks. Residues forming the hydrophobic core in the N-terminal domain are depicted as cyan sticks. (**d**) Fluorescence-based thermal stability assay.

NCS-family proteins have an N-terminal myristoylation motif, such as recoverin, and the myristoyl group is buried in the hydrophobic pocket in the Ca^2+^-free form (apo form or Mg^2+^-bound form) (Fig. 4a). Calaxin has no myristoylation motif in the N-terminus. Instead, in the crystal structure of calaxin, the hydrophobic core in the N-terminal domain interacted with hydrophobic residues (Phe191, Val195 and Leu196) on the α11 helix (Fig. 4b and c). To assess the role of the α11 helix, we prepared an α11-deletion mutant (residues 1–182) and analyzed the thermostability of WT and this mutant in the Ca^2+^-bound forms using the fluorescence-based thermal stability assay (Fig. 4d). The first derivative curve showed the melting temperature (*T*_m_) for WT and mutant calaxin. The first derivative curve of WT showed a large melting peak at 56 °C. However, the α11-deletion mutation caused a remarkable decrease in the melting peak (*T*_m_ = 28 °C). Furthermore, the first derivative curve of the mutant showed a wide positive peak near 50 °C, indicating aggregation. Although the thermal stability was decreased, the protein folding was hardly affected by the α11-deletion compared to WT (Supplementary Figs 3 and 4). The results indicate that the hydrophobic interaction of the α11 helix with the N-terminal domain contributes to the thermostability of calaxin in the Ca^2+^-bound form.

### Comparison of the structures between the Ca^2+^-bound and Mg^2+^-bound forms

To clarify the structural features in Ca^2+^-bound form, we determined the crystal structure of calaxin in the Mg^2+^-bound form and compared the structures between these forms. Although calaxin in the Mg^2+^-bound form was purified in the presence of 100 mM magnesium chloride, the crystallizing conditions contained 10 mM barium chloride. We used isothermal titration calorimetry to determine which ion was bound in calaxin. Titrations of Ba^2+^ to calaxin in the presence of Mg^2+^ showed no heat, indicating that Mg^2+^ bound to calaxin was not replaced by Ba^2+^ (Supplementary Fig. 5). In addition, the anomalous signals of Ba^2+^ ions were not observed (Supplementary Fig. 6). Therefore, we obtained the crystal structure of Mg^2+^-bound calaxin.

**Figure 5:**
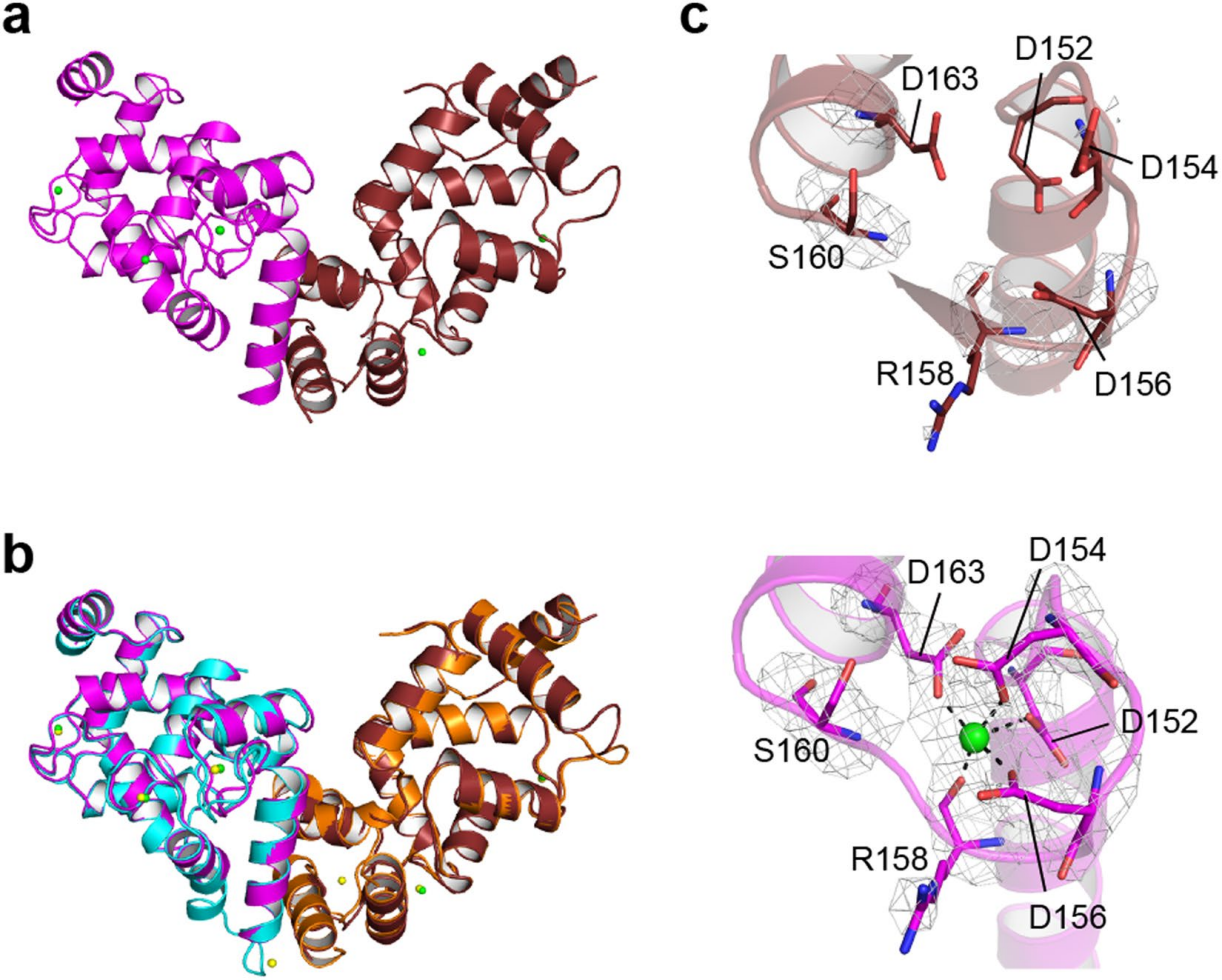
Structure of the Mg^2+^-bound form of calaxin. (**a**) Overall structure of calaxin in the Mg^2+^-bound form. The open state and closed state are depicted in magenta and ruby, respectively. Mg^2+^ ions are shown in green spheres. (**b**) Structural comparison between the Ca^2+^-bound forms (open state: cyan, closed state: orange) and Mg^2+^-bound forms (open state: magenta, closed state: ruby) of calaxin. (**c**) Enlarged views of EF3 in the open state (lower pannel) and closed state (upper panel). The *F*_o_ − *F*_c_ omit maps of the open and closed states are shown as gray meshes contoured at 3σ.

The crystal structures are similar between the Ca^2+^-bound and Mg^2+^-bound form (RMSD 0.516 Å), and the open and closed states were observed also in the Mg^2+^-bound form, supporting that Ca^2+^ is insufficient for the driving force to convert between the open and closed states (Fig. 5a and b). Mg^2+^ ions in EF1 and EF3 are liganded with octahedral coordination geometry in the open state, due to lack of the monodentate binding at −Z position as compared with Ca^2+^ ions, which is attributed to the difference of the ionic radii between Mg^2+^ and Ca^2+^. However, no electron density of Mg^2+^ was observed in EF3 of the closed state, and the side chains of the loop between E-helix and F-helix, especially Asp152 and Asp154, seem to be flexible because of poor electron density (Fig. 5c). These data indicate that the affinity of EF3 for Mg^2+^ is different between the open and closed states: Mg^2+^ prefers to bind to the open state over the closed state.

### Changes in structural transition between the Ca^2+^-bound and Mg^2+^-bound forms in solution

**Figure 6:**
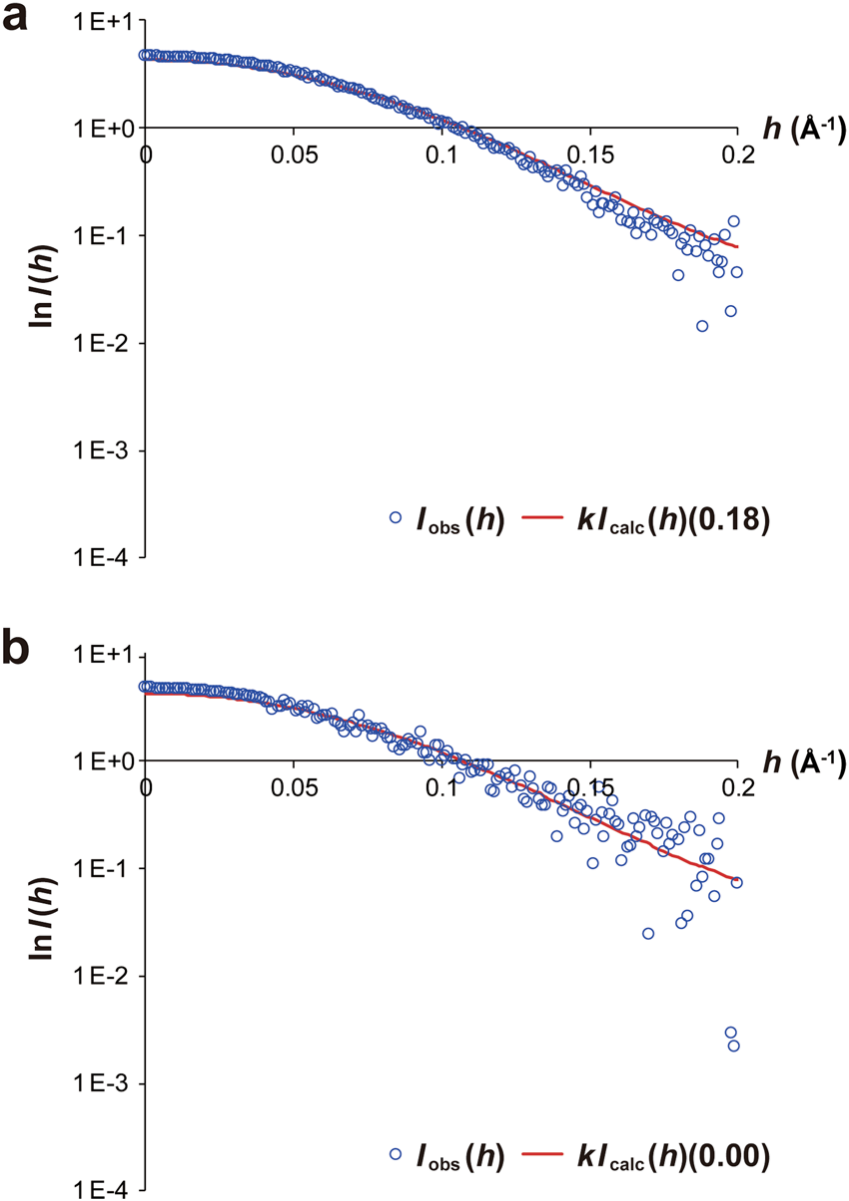
Structural transition between the open and closed state of calaxin observed in SAXS experiments. (**a**) Experimental (blue circles) and calculated (fraction of closed state = 0.18) (red line) scattering curves of Ca^2+^-bound calaxin. (**b**) Experimental (blue circles) and calculated (fraction of closed state = 0.00) (red line) scattering curves of Mg^2+^-bound calaxin.

To evaluate the conformation of calaxin in the Ca^2+^ and Mg^2+^-bound forms in solution, we carried out small-angle X-ray scattering (SAXS) experiments in the presence of Ca^2+^ or Mg^2+^ ion. Based on the Guinier plots (Supplementary Fig. 7), the radius of gyration (*R*_*g*_) was 19.3 and 20.2 Å for Ca^2+^ and Mg^2+^-bound forms, respectively. These data show that calaxin exists a monomer in the both states. We further estimated the population of open and closed conformers under equilibrium in solution by fitting the intrinsic scattering curves, which were theoretically calculated with crystal structures of the two states of calaxin, to experimental scattering curves of the Ca^2+^ and Mg^2+^-bound calaxin (Fig. 6). Fraction of the open state with the lowest *R* factor was 0.18 in the presence of Ca^2+^ ion, which shows that calaxin adopts the closed and open sates with abundance ratio of 18% and 82% in the Ca^2+^-bound form, respectively. In contrast, the existing rate of Mg^2+^-bound calaxin was limited to only open state (100%). These results suggest that the Ca^2+^ binding functions to shift open-closed structural transition toward the closed state. In addition, the open state may be stabilized by a selective binding of Mg^2+^ to EF3 in the absence of Ca^2+^ ion.

## Discussion

**Figure 7:**
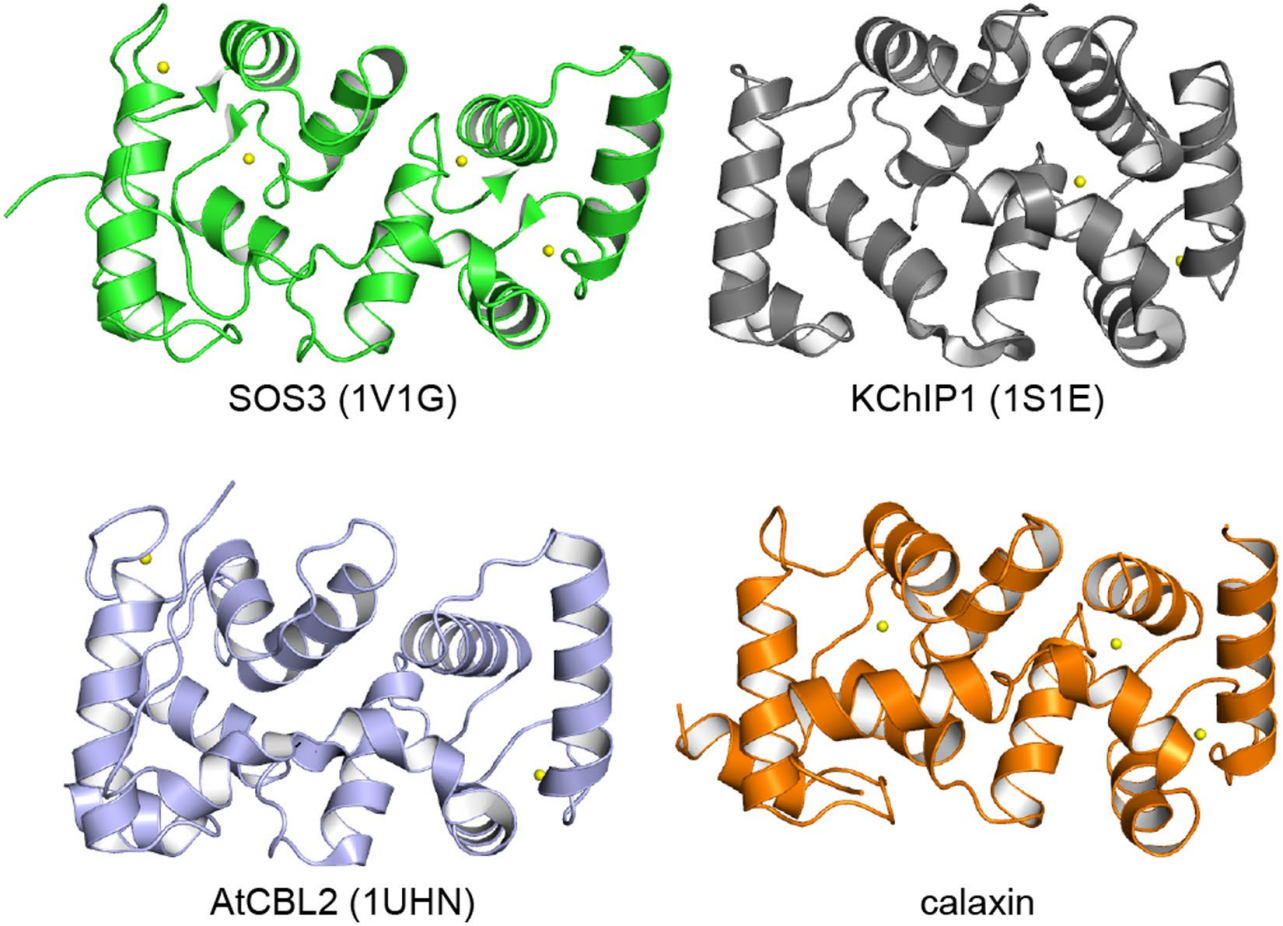
Crystal structures of Ca^2+^-bound NCS-family proteins. Structural comparison among NCS-family proteins, SOS3 (PDB ID: 1V1G), KChIP1 (PDB ID: 1S1E), AtCBL2 (PDB ID: 1UHN) and calaxin in the closed state. Yellow spheres indicate Ca^2+^ ions.

In the typical target-recognition mechanism of NCS-family proteins, Ca^2+^ binding to a protein induces the extrusion of the N-terminal myristoyl group, which sequesters the hydrophobic groove and covers up the target binding site in the closed state (Supplementary Fig. 8a). This conformational change results in the exposure of residues interacting with the target proteins^26–29^. However, there is no myristoylation motif in the N-terminus of calaxin^24^. Several structures of NCS-family proteins have also been determined and are similar to the structure of calaxin (Fig. 7). The C-terminal regions of the NCS-family proteins provide the surface to receive the target protein in the Ca^2+^-bound form, such as KChIP1^35^ and AtCBL2^36^ (Supplementary Fig. 8b and c). In contrast to these typical NCS-family proteins, calaxin shows an open-closed transition state in the Ca^2+^-bound form. In the closed state, the exposure of the hydrophobic surface of calaxin is reduced by the movement of the α7 and α8 helices instead of the movement of the N-terminal myristoyl group or C-terminal region in the other NCS-family proteins. Typical NCS-family proteins receive the α-helical structure of target proteins with the induced surfaces of the Ca^2+^-bound forms. Therefore, the Ca^2+^-induced surface in the C-terminal domain of calaxin may participate the interaction with dynein, whose structure is largely composed of α-helices^37^.

EF-hand proteins are known to change the conformation and metal-binding pattern between the Ca^2+^-bound and Mg^2+^-bound forms^30,38,39^. However, calaxin in the Mg^2+^-bound form shows a similar structure to that of the Ca^2+^-bound form, indicating that the conformational transition between the Ca^2+^-bound and Mg^2+^-bound forms is insignificant and that the effect of the crystal packing may surpass that of the conformational transition. Interestingly, we found that EF3 has a lower affinity for Mg^2+^ in the closed state due to incompatible arrangements of the acidic residues (Fig. 5c), whereas Ca^2+^ induces the same arrangements of the EF3 both in the closed and open states. These structural differences in the Ca^2+^- and Mg^2+^-bound forms indicate the possibility that calaxin in the Ca^2+^-bound form is more compatible with the conformational change than the Mg^2+^-bound form. Moreover, SAXS data supports that Ca^2+^-binding promotes the open-closed structural transition toward the closed state (Fig. 6). A transient [Ca^2+^]_i_ is increased up to 10^−4^ M in the process of the directional changes of sperm movement^21^. In addition to the Ca^2+^ binding to the EF-hands causing structural transition, the interaction with outer-arm dynein may be essential to complete the conformational change of calaxin.

In the crystal structure of AtCBL2, which is a member of the NCS protein family, the helices in the C-terminal domain form the hydrophobic crevice and accommodate the regulatory domain of AtCIPK14 (Supplementary Fig. 8c)^36^. Furthermore, Ca^2+^ ions are bound by AtCBL2 with and without AtCIPK14, supporting that a conformational transition between the ligand-bound and ligand-free forms can occur in other NCS-family proteins, including calaxin, when the proteins adopt their Ca^2+^-bound states. However, the binding patterns of Ca^2+^ ions are different between the ligand-bound and ligand-free forms of AtCBL2. All four EF-hands are filled with Ca^2+^ ions in the ligand-bound form, whereas Ca^2+^ ions bind only to the first and fourth EF-hands in ligand-free form. On the other hand, opposite Ca^2+^ binding patterns are shown in the structure of SOS3 complexed with SOS2. SOS3 in the SOS2-bound form binds Ca^2+^ ions only at the first and fourth EF-hands, although all four EF-hands are occupied with Ca^2+^ ions in the SOS2-unbound form^40^. Calaxin adopts both its open state and closed state in the same Ca^2+^-binding pattern in which three Ca^2+^ ions are coordinated to EF1, EF2 and EF3, indicating that its open-closed transition is independent of the Ca^2+^-binding pattern.

Dynein family proteins use a common principle to generate movement in which they bind to their track, undergo a force-producing conformational change, are released from the track and then return to their original conformation. Therefore, corresponding to the action of dynein family members, conformational change will help calaxin recognize dynein effectively. Although the core structures constituted by four EF-hands (α2 through α9) are similar among NCS-family proteins, their C-terminal helices and loops of each protein adopt various conformations (Supplementary Fig. 9). This indicates that the conformational differences in the C-terminal helices are involved in the selectivity of NCS-family proteins toward associating partners. Also, our fluorescence-based thermal stability assay demonstrated that the C-terminal helix of calaxin contributes the conformational stability by interaction with the hydrophobic core in the N-terminal domain. Although further demonstration requires the identification of the target binding region of dynein and subsequent structural analyses of calaxin complexed with dynein or its target region, our findings provides a plausible conformational change consistent with outer-arm dynein mechanism of the calaxin-mediated regulation of dynein induced by a transient [Ca^2+^]_i_ increase.

## Materials and Methods

### Expression and Purification of Ca^2+^-bound and Mg^2+^-bound Calaxin

For the preparation of calaxin (UniProt ID: Q8T893), the calaxin gene was cloned at *Nde*I and *Bam*HI sites of the pET-28a vector (Novagen), and the vector was transformed to the *Escherichia coli* K12 strain KRX cells (Promega). The cells were grown in Lysogeny Broth (LB) medium containing 30 μg mL^−1^ kanamycin at 37 °C. After the addition of 0.1% L-rhamnose (Wako) at an optical density at 600 nm (OD_600_) of 0.6, the cell culture was further incubated at 20 °C for 18 h. The cells were harvested by centrifugation at 2,290 × *g* for 15 min at 4 °C. The pellet was resuspended in sonication buffer containing 50 mM Tris-HCl (pH 8.0), 300 mM NaCl and 10 mM imidazole. After sonication, the suspension was centrifuged at 40,000 × *g* for 30 min at 4 °C to remove cell debris and insoluble fractions. The supernatant was loaded onto a His-tag affinity column prepared by filling a 20-mL chromatography column (BioRad) with Ni Sepharose 6 Fast Flow (GE Healthcare). After the column was washed with washing buffer containing 50 mM Tris-HCl (pH 8.0), 300 mM NaCl and 50 mM imidazole, calaxin was eluted with elution buffer containing 50 mM Tris-HCl (pH 8.0), 300 mM NaCl and 250 mM imidazole. Thrombin was added to the eluted protein to remove the N-terminal His-tag, and the protein solution was then dialyzed overnight against chelate buffer containing 50 mM Tris-HCl (pH 8.0), 1 mM DTT and 1 mM EDTA. The solution was further dialyzed against 50 mM Tris-HCl (pH 8.0), 1 mM DTT and 1 mM CaCl_2_ to prepare the calcium binding protein. The protein was purified using a Resource Q (GE Healthcare) anion exchange column equilibrated with equilibration buffer containing 25 mM Tris-HCl (pH 8.0), 1 mM DTT and 1 mM CaCl_2_ and was eluted by increasing the NaCl concentration from 0 to 500 mM in the equilibration buffer. For the preparation of Mg^2+^-bound calaxin, the protein solution was dialyzed against 20 mM Tris-HCl (pH 8.0), 20 mM MgCl_2_ and 1 mM DTT. Furthermore, the dialyzed Mg^2+^-bound calaxin was eluted with a 0 to 300-mM NaCl gradient from a Resource Q column, followed by buffer exchange with a Superdex 75 10/300 HR (GE Healthcare) column using 10 mM MES-NaOH (pH 6.0), 100 mM MgCl_2_, 150 mM NaCl and 1 mM DTT.

### Crystallization, Data Collection and Structure Determination

Crystallization of the Ca^2+^-bound protein was performed using the sitting drop vapor diffusion method. Each sitting drop was prepared by mixing the protein solution (0.75 μL) with the equal volume of reservoir solution (0.75 μL) containing 0.1 M MES (pH 6.4), 0.2 M calcium acetate and 17.5% PEG8000 and then was equilibrated against the reservoir solution at 20 °C. The concentration of protein for crystallization was 10 mg mL^−1^, and 24% ethylene glycol was used as the cryoprotectant. The X-ray diffraction data were collected with an in-house X-ray diffractometer equipped with an FR-E SuperBright X-ray generator and an R-AXIS VII imaging plate. For crystallographic phasing, the crystal was soaked in a solution containing 0.1 M MES (pH 6.2), 10 mM SmCl_3_ and 16% PEG8000, and the single anomalous diffraction (SAD) data were collected at the samarium peak wavelength of 1.6 Å on the BL-17A beamline at Photon Factory (Tsukuba, Japan).

The collected data sets were indexed and integrated using XDS^41^, and were scaled using XSCALE^41^. Experimental phasing was performed with the SAD data and the program package autoSHARP^42^. The initial Model was automatically built with ARP/wARP^43^ in the CCP4 suite^44^. After automatic modeling, manual model building was carried out with Coot^45^, and Refmac5^46^ was used for the refinement of the obtained model. To determine the structure of Ca^2+^-bound protein, the molecular replacement method was carried out using Molrep^47^ and the samarium-bound structure as a template. Model building and refinement were performed using Coot and Refmac5, respectively. The quality of the models was verified by PROCHECK^48^. The images of the protein structure were created by PyMol (http://www.pymol.org/). The sequence alignment and visualization were performed using ClustalW and ESPript, respectively.

Crystallization of the Mg^2+^-bound protein was also performed using the sitting drop vapor diffusion method at 20 °C by mixing 0.3 μL of 12 mg mL^−1^ protein solution with 0.3 μL of reservoir solution (containing 0.2 M ammonium sulfate, 0.1 M MES, pH 6.3, 26% (w/v) PEG 5000 MME and 10 mM barium chloride). The X-ray diffraction data were collected at a wavelength of 1.0 Å at Photon Factory beamline AR-NW12A. The data were indexed and integrated with XDS^41^, and were scaled with AIMLESS^49^. The crystal structure of Mg^2+^-bound calaxin was determined by Molrep using the structure of the Ca^2+^-bound calaxin without Ca^2+^ ions as a template model. Coot was used for model building, and Phenix.refine^50^ and Refmac5 were used for refinement. Twin refinement was applied because twinning fraction of structure factor data for Mg^2+^-bound calaxin was 0.155.

### Preparation of Mutant Proteins

Two mutant proteins (E118A and D163A) were prepared for isothermal titration calorimetry experiments. The expression vectors were created using PrimeSTAR Max DNA Polymerase (Takara), the expression vector for wild-type protein as a template and primers (Supplementary Table 2). The protein expression and purification were carried out with the same procedure as that of the wild type.

### Isothermal Titration Calorimetry (ITC) Experiments

ITC experiments were carried out using an iTC_200_ calorimeter (GE Healthcare) at 4 °C. The protein solutions were prepared in aqueous buffer containing 25 mM MOPS-KOH (pH 7.8), 1 mM DTT and 200 mM NaCl. Calcium titrations were carried out with 1 mM CaCl_2_ and a 50-μM protein solution. The experiments were carried out with each injection consisting of 1.5 μL of the titrant against 200 μL of the protein solution. Data analysis was performed using Origin 7 software. The titration curve of the D163A mutant was fitted using a two sequential binding model. Barium titrations were carried out using 0.8 mM BaCl_2_ and a 35-μM calaxin solution in the buffer containing 25 mM MOPS-KOH (pH 7.8), 1 mM DTT, 200 mM NaCl and 1 mM MgCl_2_. Data analysis was conducted in the same way as that for Ca^2+^ titrations.

### Fluorescence-based thermal stability assay

The assay was carried out using the CFX Connect Real-Time System (Bio-Rad) as previously described^51^. The sample solutions were prepared by mixing 2.22 μL of 100× SYPRO Orange (Life Technologies) and 20 μL of 0.3 mg/mL calaxin solution (20 mM Tris-HCl, pH 8.0, 150 mM NaCl, 1 mM DTT and 1 mM CaCl_2_). Each sample was heated from 20 to 95 °C in increments of 0.5 °C.

### Circular dichroism measurements

Wild type calaxin and its mutants (E118A, D163A and the α11-deletion mutant) were analyzed by circular dichroism to compare their secondary structures. The decalcified WT, E118A and D163A samples (11 μM) were prepared in a buffer of 25 mM MOPS-KOH (pH 7.8) and 1 mM DTT, and the α11-deletion mutant (11 μM) was prepared in a buffer containing 20 mM Tris-HCl (pH 8.0), 150 mM NaCl, 1 mM DTT and 1 mM CaCl_2_. The measurements were performed at room temperature by using a Jasco J-720 spectropolarimeter (WT, E118A and D163A) and a Jasco J-820 spectropolarimeter (the α11-deletion mutant) with a quartz cuvette of 0.1 cm path length. CD spectra were recorded and analyzed between 200–260 nm.

### Small-angle X-ray scattering experiments and analyses

The SAXS experiments were carried out on beamline BL-10C at the Photon Factory. All data were collected using X-ray of wavelength of 1.488 Å with a PILATUS3 2 M detector (Dectris) and processed with the FIT2D program (http://www.esrf.eu/computing/scientific/FIT2D/). The sample-to-detector distance was 1.0 m. Equilibrium experiments were performed at 25 °C. The SAXS intensities were accumulated for a total of 30 s by repeating the measurements for a period of 1.0 s each time in order to ensure enough statistical precision. X-ray scattering data were obtained from protein and the corresponding buffers. The scattering data of the buffers were subtracted from those of the protein solutions. X-ray scattering data were analyzed by Guinier approximation, as assuming an exponential dependence of the scattering intensity on *h*^2^, where *h* = 4πsin*θ*/λ and *θ* is half of the scattering angle^52^. *R*_*g*_ and zero angle scattering intensity *I*(0) were determined using Guinier approximation^52^. Molecular weight was determined from *I*(0) by measuring bovine serum albumin (BSA) as a calibration standard^53^. The sample concentrations of calaxin were 0.25, 0.5, 1.0 and 1.75 mg/mL for the Ca^2+^-bound form and 0.25, 0.5, 1.0 and 2.0 mg mL^−1^ for the Mg^2+^-bound form. The samples of the Ca^2+^- and Mg^2+^-bound forms were prepared in 20 mM Tris-HCl (pH 8.0), 150 mM NaCl and 1 mM DTT containing 10 mM CaCl_2_ and MgCl_2_, respectively.

The intrinsic scattering intensities of open and closed conformers were theoretically calculated from atomic coordinates of their crystal structures by CRYSOL^54^ program for default parameter settings. The scattering intensity *I*_calc_(*h*) of equilibrium mixture of open and closed forms with a ratio of (1 − *α*): *α* was expressed as:

$${I}_{calc}(h)=(1-\alpha ){I}_{open}(h)+\alpha {I}_{closed}(h)\,$$

where *I*_open_(*h*) and *I*_closed_(*h*) are intrinsic scattering intensities of open and closed conformers, respectively. We evaluated optimal value of *α*, fraction of the closed conformer under equilibrium in solution, which yielded the lowest *R* factor such as:

$$R=\frac{\sum _{h}|{I}_{obs}(h)-k{I}_{calc}(h)|{h}^{2}}{\sum _{h}{I}_{obs}(h){h}^{2}}$$

where *I*_obs_(*h*) is the experimental scattering intensity, and *k* is the scaling factor between observed and calculated scattering intensities as:

$$k=\frac{\sum _{h}{I}_{obs}(h){I}_{calc}(h){h}^{2}}{\sum _{h}{I}_{calc}{(h)}^{2}{h}^{2}}$$

### Data Availability

The atomic coordinates have been deposited in the Protein Data Bank, www.pdb.org (PDB ID code 5X9A and 5YPX).

## Electronic supplementary material

Supplementary Information
