# Supplementary material for: Crystal structure of a Ca2+-dependent regulator of flagellar motility reveals the open-closed structural transition
Source: Sci Rep. 2018 Jan 31;8:2014. doi: 10.1038/s41598-018-19898-7 (PMC5792641; doi:10.1038/s41598-018-19898-7)
Supplement: Supplementary file 1 — Supplementary Information [file 41598_2018_19898_MOESM1_ESM.pdf]

Supplementary Information for

Crystal structure of a Ca^{2+}-dependent regulator of flagellar motility reveals the open-closed structural transition

Tomoki Shojima[1], Feng Hou[1], Yusuke Takahashi[1], Yoshitaka Matsumura[2], Masahiko Okai[1], Akira Nakamura[1], Katsutoshi Mizuno[3], Kazuo Inaba[3], Masaki Kojima[2], Takuya Miyakawa[1], and Masaru Tanokura[1]*

Affiliations:

[1]Department of Applied Biological Chemistry, Graduate School of Agricultural and Life Sciences, The University of Tokyo, 1-1-1 Yayoi, Bunkyo-ku, Tokyo 113-8657, Japan

[2]Laboratory of Bioinformatics, School of Life Sciences, Tokyo University of Pharmacy and Life Science, Hachioji, Japan

[3]Shimoda Marine Research Center, University of Tsukuba, Shizuoka 415-0025, Japan

*Corresponding author. Email: amtanok@mail.ecc.u-tokyo.ac.jp

Contents

Supplementary Table 1: Data collection and refinement statistics.

Supplementary Table 2: Primers used in this study.

Supplementary Figure 1: Multiple sequence alignment of the EF-hand motifs of NCS-family proteins,

Supplementary Figure 2: Crystal-packing interaction between the hydrophobic surfaces in the open state (cyan) and α helices in the closed state (orange).

Supplementary Figure 3: Circular dichroism studies of wild-type calaxin (a), E118A (b) and D163A (c) in the apo forms.

Supplementary Figure 4. Circular dichroism study of the α11-deletion mutant.

Supplementary Figure 5: Isothermal titration calorimetry of Ba^{2+} binding to calaxin in the Mg^{2+}-bound form.

Supplementary Figure 6. Anomalous difference Fourier map of Mg^{2+}-bound calaxin.

Supplementary Figure 7. Guinier plots of Ca^{2+} and Mg^{2+}-bound calaxins at different concentrations.

Supplementary Figure 8: Conformational change of NCS-family proteins upon Ca^{2+} binding or ligand binding.

Supplementary Figure 9: Structural comparison among NCS-family proteins.

Supplementary Table 1. Data collection and refinement statistics.

	Sm calaxin	Ca calaxin	Mg calaxin
**Data collection**			
Space group	$P4_3$	$P4_3$	$P4_3$
Unit Cell *a*, *b*, *c* (Å)	65.0, 65.0, 113.3	65.5, 65.5, 113.5	65.3, 65.3, 113.2
Wavelength (Å)	1.6000	1.5418	1.0000
Resolution (Å)[a]	20.0–2.00 (2.05–2.00)	20.0–1.85 (1.90–1.85)	46.1–2.64 (2.77–2.64)
R_{meas}[a,b] (%)	5.5 (36.1)	4.4 (37.9)	12.4 (75.5)
$CC_{1/2}$	99.8 (89.5)	100 (88.3)	99.7 (81.3
$<I/\sigma(I)>$[a]	16.6 (3.5)	32.1 (3.8)	17.2 (3.2)
Completeness (%)[a]	97.1 (92.1)	98.9 (89.0)	100 (99.9)
Redundancy[a]	3.7 (3.5)	6.6 (3.0)	7.6 (7.5)
Reflections/unique	223469/60617	268522/40393	107108/14030
Wilson B factor (Å^2)	27.1	17.2	26.5
**Refinement**			
Resolution (Å)	–	19.8–1.85 (1.90–1.85)	42.8–2.64 (2.71–2.64)
R_{work}/R_{free}[c] (%)	–	17.7/21.9 (23.0/29.3)	16.3/21.2 (21.4/33.6)
No. Protein atoms	–	3170	3115
No. non-protein atoms			
Metal	–	7	5
Solvent	–	225	21
Others	–	4	0
Average *B*-factor	–		
Protein		25.8	50.0
Ion		22.3	35.7
Water		29.9	32.0
Root mean square deviations			
Bond lengths (Å)	–	0.020	0.015
Bond angles (Å)	–	1.988	1.761
Ramachandran plot			
Most favorable (%)	–	98.42	95.84
Allowed (%)	–	1.58	4.16
Disallowed (%)	–	0.00	0.00

[a]Values in parentheses are for the highest resolution shell.

[b]$R_{meas} = \sum_{hkl} [\{n/(n-1)\}^{1/2}(\sum_i |I_i - \langle I\rangle|)/\Sigma_i |I_i|]$, where I_i is the *i*th intensity measurement of reflection *hkl*, including symmetry-related reflections, and $\langle I\rangle$ is its average.

[c]R_{free} was calculated using 5% of the reflections omitted from the refinement.

Supplementary Table 2. Primers used in this study.

Primer	Sequence
E118A_forward	5’ -CGTGAAGCAATGTTTCAAATGTTGAAGACATG- 3’
E118A_reverse	5’ -AAACATTGCTTCACGAGAAATGTAACCATCACC- 3’
D163A_forward	5’ -AAAAAGCTTTCAAAGATGCCGTTCTTATTGAACC- 3’
D163A_reverse	5’ -CTTTGAAAGCTTTTTTTGATAATCTGCTGTCATGA- 3’

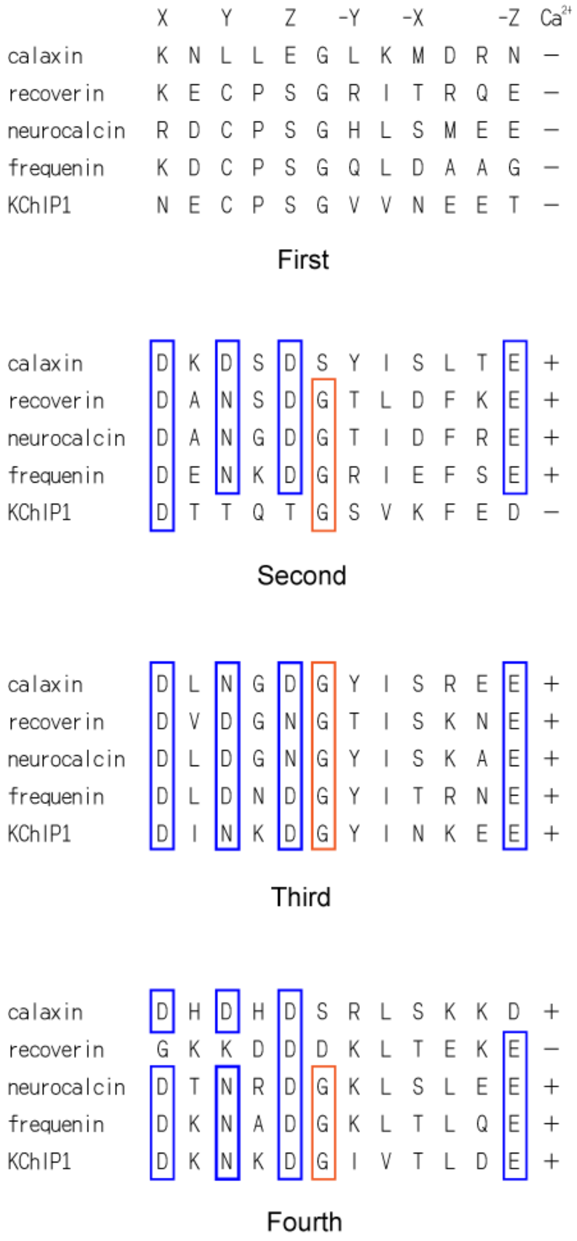

Supplementary Figure 1. Multiple sequence alignment of the EF-hand motifs of NCS-family proteins, including calaxin, recoverin, neurocalcin, frequenin and KChIP1. Highly conserved residues are highlighted by the blue box. Gly residues between positions Z and –Y are indicated by the orange box. The Ca^{2+}-binding ability of EF-hand motifs is shown in the rightmost column (Ca^{2+}).

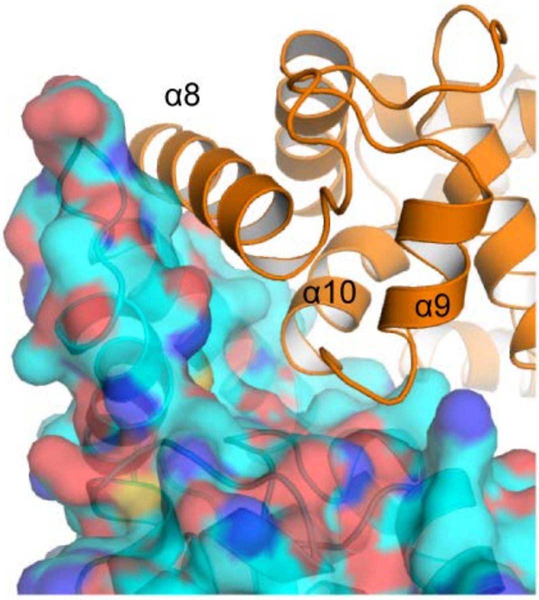

Supplementary Figure 2. Crystal-packing interaction between the hydrophobic surfaces in the open state (cyan) and α helices in the closed state (orange).

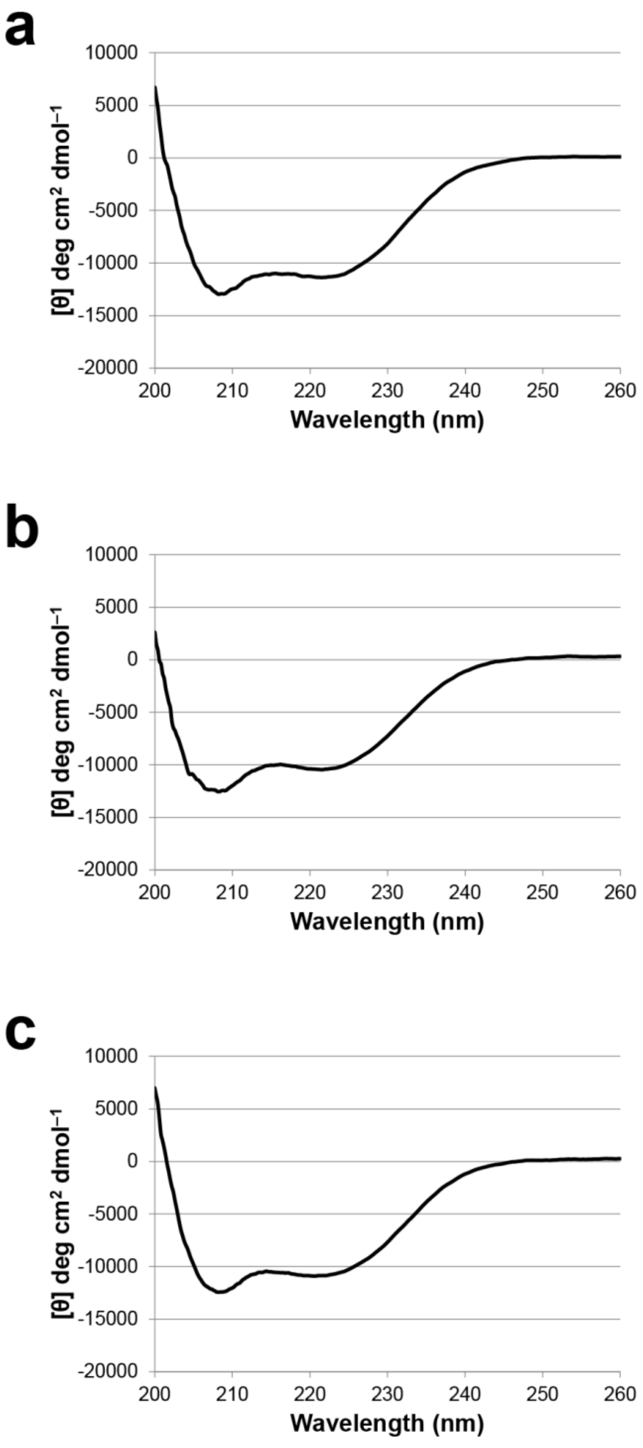

Supplementary Figure 3. Circular dichroism studies of wild-type calaxin (a), E118A mutant (b) and D163A mutant (c) in the apo forms.

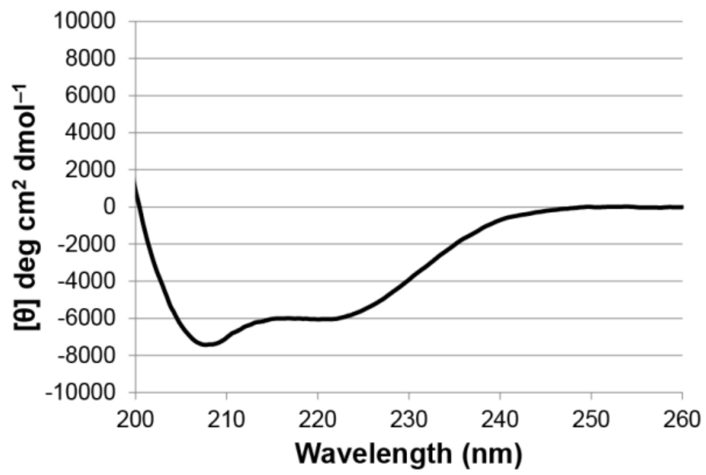

Supplementary Figure 4. Circular dichroism study of the α11-deletion mutant.

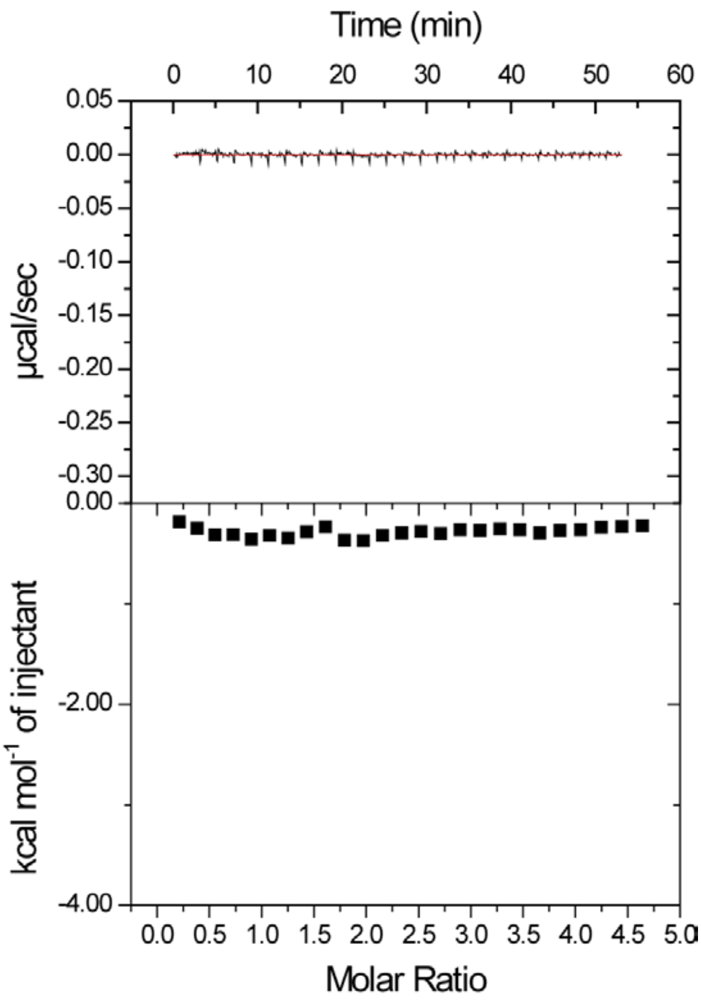

Supplementary Figure 5. Isothermal titration calorimetry of Ba^{2+} binding to calaxin in the Mg^{2+}-bound form.

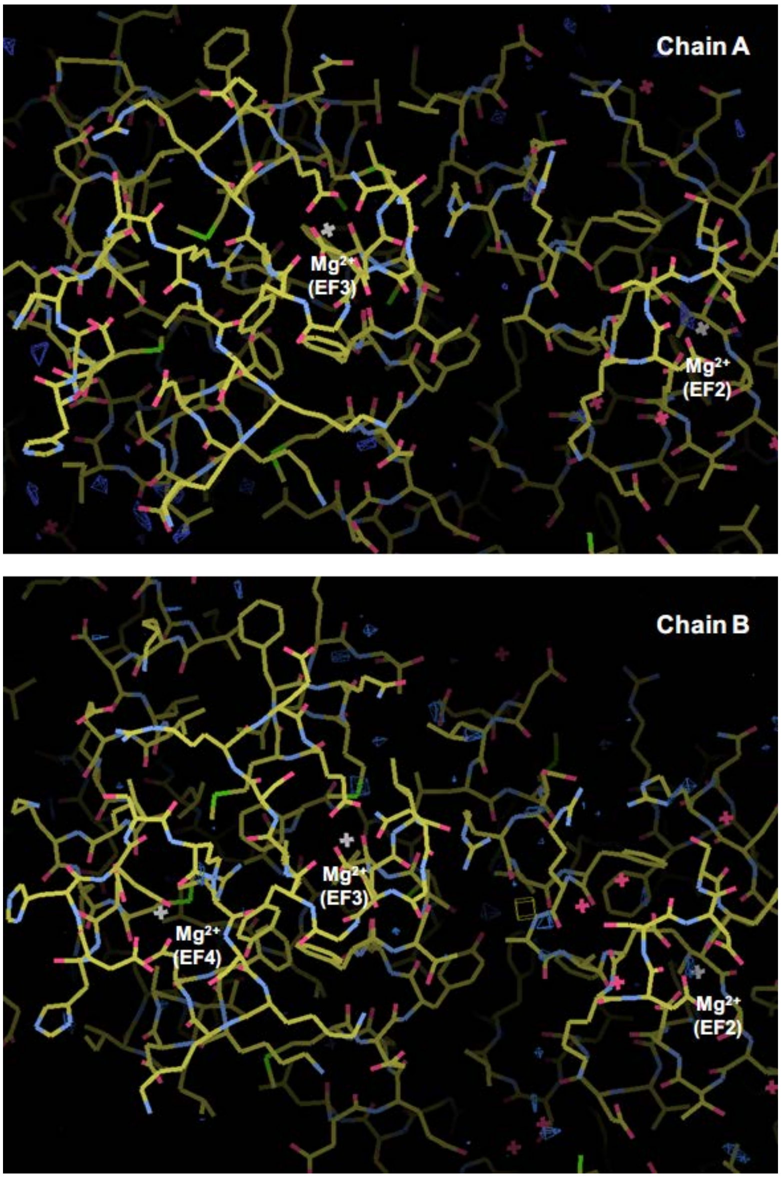

Supplementary Figure 6. Anomalous difference Fourier map of Mg^{2+}-bound calaxin. Mesh diagrams represent the anomalous difference Fourier map (blue, 3.0σ).

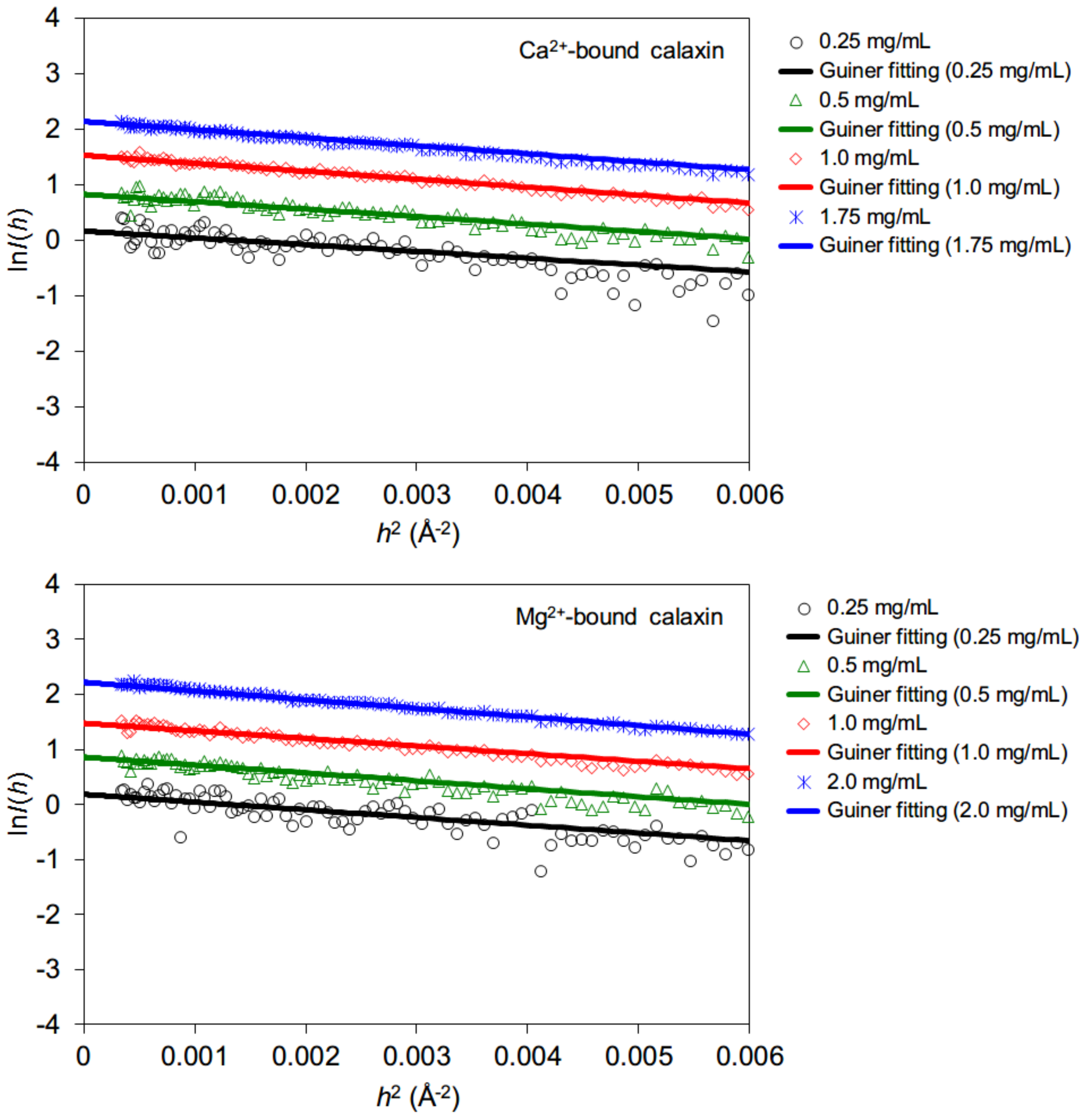

Supplementary Figure 7. Guinier plots of Ca^{2+} and Mg^{2+}-bound calaxins at different concentrations.

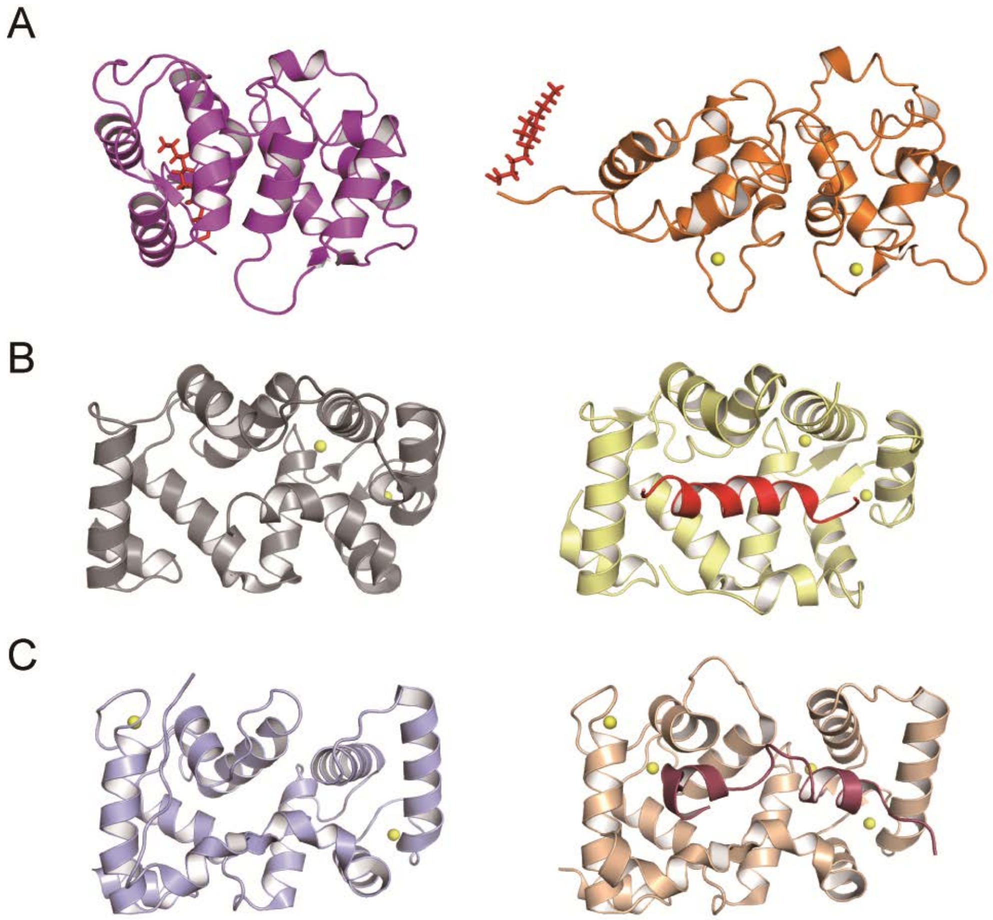

Supplementary Figure 8. Conformational change of NCS-family proteins upon Ca^{2+} binding or ligand binding. (**A**) Solution structures of N-myristoylated recoverin in the Ca^{2+}-free form (left, PDB ID: 1IKU) and Ca^{2+}-bound form (right, PDB ID: 1JSA). Ca^{2+} ions and myristoyl groups are shown as yellow spheres and red sticks, respectively. (**B**) Crystal structures of KChIP1 in the ligand-free form (left, gray, PDB ID: 1S1E) and Kv4.3-bound form (right, pale yellow, PDB ID: 2I2R). The N-terminal helix of Kv4.3 is depicted as a red ribbon (residues 3–20). (**C**) Crystal structures of AtCBL2 in the ligand-free form (left, light blue, PDB ID: 1UHN) and AtCIPK14-bound form (right, wheat, PDB ID: 2ZFD). The regulatory domain of AtCIPK14 associating with the hydrophobic surface of AtCBL2 is depicted in raspberry (residues 308–332).

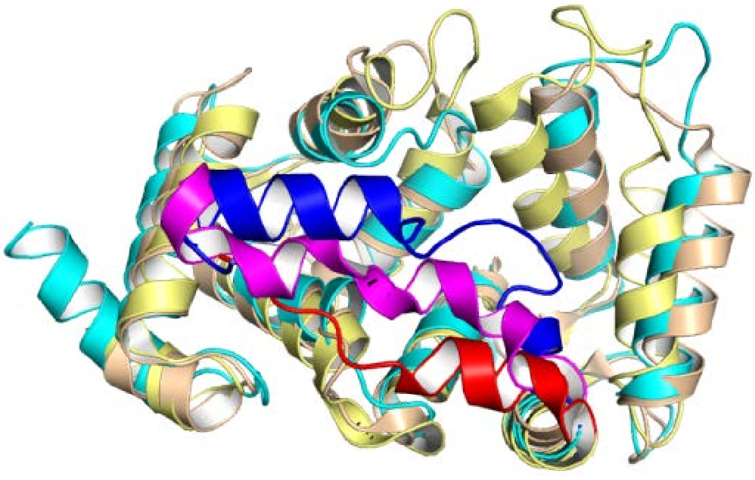

Supplementary Figure 9. Structural comparison among NCS-family proteins. Crystal structures of calaxin in the open state (cyan and blue), KChIP1 (pale yellow and red) and AtCBL2 (wheat and magenta) are depicted in ribbon models. Blue, red and magenta ribbons represent the C-terminal helices of each protein.
